# Supplementary material for: A model of head direction and landmark coding in complex environments
Source: PLoS Comput Biol. 2021 Sep 27;17(9):e1009434. doi: 10.1371/journal.pcbi.1009434 (PMC8496825; doi:10.1371/journal.pcbi.1009434)
Supplement: S1 Appendix — (DOCX) [file pcbi.1009434.s001.docx]

**S1 Appendix. Synaptic connections.**

For plastic connections, unless specified otherwise, classic Hebbian learning is used,

$$\begin{aligned} \boldsymbol{W}^{\left( j \right)}\left( t+\Delta t \right)=\boldsymbol{W}^{\left( j \right)}\left( t \right)+\eta_{j}\boldsymbol{f}\left( t \right){\boldsymbol{f}^{\left( j \right)}\left( t \right)}^{T} ,\#\left( \mathrm{AUTONUM} \right) \end{aligned}$$

where $\eta_{j}$ is the learning rate corresponding to the connection with the $j$th input layer, and $\boldsymbol{f}\left( t \right)$ is the firing-rate vector of the output layer. During the testing phase, we set $\eta_{j}=0$ to fix synaptic connections.

For the classic Hebbian learning, weights are normalized to ensure the stability of learning dynamics with this learning rule. More specifically,

$$\begin{aligned} \boldsymbol{w}_{l}^{\left( j \right)}\left( t+\Delta t \right)\to\max\left\{ \frac{w_{\max}^{(j)}}{\left\| \boldsymbol{w}_{l}^{\left( j \right)}\left( t+\Delta t \right) \right\|_{2}}, 1 \right\}\boldsymbol{w}_{l}^{\left( j \right)}\left( t+\Delta t \right) \forall l ,\#\left( \mathrm{AUTONUM} \right) \end{aligned}$$

where $\left\| \cdot\right\|_{2}$ is the two-norm for calculating the total connection, $w_{\max}$ is the maximum total connection strength, and $\boldsymbol{w}_{l}$ is the $l$th row vector (i.e. synaptic connections targeting the $l$th output neuron) of the weight matrix $\boldsymbol{W}$.

Non-plastic connections include 1-to-1 feedforward projections and inhibitory connections, requiring the same number of neurons $N$ both in the input and output layer. In the case of self-inhibition, the input and the output layer are the same. The 1-to-1 feedforward projection could be written as an $N\times N$ identity matrix $\boldsymbol{I}\left( N \right)$. The inhibitory connection includes two types of inhibition: global inhibition

$$\begin{aligned} \boldsymbol{I}_{\mathrm{GI}}\left( N \right)\boldsymbol{=}\frac{\boldsymbol{J}\left( N \right)}{\sqrt{N}} ;\#\left( \mathrm{AUTONUM} \right) \end{aligned}$$

and lateral inhibition

$$\begin{aligned} \boldsymbol{I}_{\mathrm{LI}}\left( N \right)\boldsymbol{=}\frac{\boldsymbol{J}\left( N \right)\boldsymbol{-I}\left( N \right)}{\sqrt{N-1}} .\#\left( \mathrm{AUTONUM} \right) \end{aligned}$$

Here $\boldsymbol{I}\left( N \right)$ is an $N\times N$ identity matrix and $\boldsymbol{J}\left( N \right)$ is an $N\times N$ all-ones matrix. These connections are kept stable at all times, i.e. they are not subject to learning. See S2 Table for a summary of all neural connections used in the paper.
