# Supplementary material for: A model of head direction and landmark coding in complex environments
Source: PLoS Comput Biol. 2021 Sep 27;17(9):e1009434. doi: 10.1371/journal.pcbi.1009434 (PMC8496825; doi:10.1371/journal.pcbi.1009434)
Supplement: S2 Appendix — (DOCX) [file pcbi.1009434.s002.docx]

**S2 Appendix. HD attractor with a single ring.**

We use a single ring HD attractor to generate HD information regulated by idiothetic information (HD angular velocity and acceleration) from the vestibular system, with the same mechanisms as [1].

The HD attractor consists of $N_{\mathrm{HD}}$ HD neurons with different preferred directions initially locked to the allocentric frame (i.e. NESW) as $\boldsymbol{\theta}=\left\{ \theta_{l}=-180+(l-1)\Delta\theta| 1\leq l\leq N_{\mathrm{HD}} \right\}$, where $N_{\mathrm{HD}}\Delta\theta=360$, $\Delta\theta$ is the angular sampling gap, $\theta_{l}=0$ stands for East, and $\theta_{l}=90$ stands for North.

The attractor contains a simplified self-connection among these neurons, with both excitatory and inhibitory connections merged into a single rotation-independent synaptic weight matrix $\boldsymbol{W}_{\mathrm{HD}}$. Therefore, the neural dynamics on the HD attractor without external inputs can be written as

$$\begin{aligned} \tau_{\mathrm{HD}}\frac{d\boldsymbol{a}_{\mathrm{HD}}}{dt}=-\boldsymbol{a}_{\mathrm{HD}}+\frac{g_{\mathrm{HD}}}{n_{\mathrm{HD}}}\boldsymbol{W}_{\mathrm{HD}}\boldsymbol{f}_{\mathrm{HD}} ,\#\left( \mathrm{AUTONUM} \right) \end{aligned}$$

with its gain factor $g_{\mathrm{HD}}=n_{\mathrm{HD}}$ for simplicity. In addition, the activation function for the HD attractor is specifically given as a modified sigmoid function $\sigma_{\mathrm{HD}}\left( x \right)=\left( \exp\left( -2\beta_{\mathrm{HD}}\left( x-\alpha_{\mathrm{HD}} \right) \right)+1 \right)^{-1}$.

The self-connection is pre-wired to sustain the presumed ideal activation $\tilde{\boldsymbol{a}}$ and its related firing pattern $\tilde{\boldsymbol{f}}=\sigma\left( \tilde{\boldsymbol{a}} \right)$, indicating that the HD attractor should hold $\tilde{\boldsymbol{f}}$ as a stable firing pattern without extra current as external inputs. We use a scaled von Mises distribution [2] as the circular normal distribution for $\tilde{\boldsymbol{f}}$, i.e.

$$\begin{aligned} \tilde{\boldsymbol{f}}\left( \tilde{\kappa}, \tilde{f}_{\max} \right)=\frac{\tilde{f}_{\max}}{e^{\tilde{\kappa}}}e^{\tilde{\kappa}\cos\left( \frac{\pi}{180}\boldsymbol{\theta} \right)},\#\left( \mathrm{AUTONUM} \right) \end{aligned}$$

where $\tilde{\kappa}$ is the encoding precision and $\tilde{f}_{\max}$ is the expected maximum firing rate.

More specifically, we give an approximate solution for $\boldsymbol{W}_{\mathrm{HD}}$ of

$$\begin{aligned} \tilde{\boldsymbol{a}}={\tilde{\boldsymbol{W}}}_{\mathrm{HD}}\tilde{\boldsymbol{f}},\#\left( \mathrm{AUTONUM} \right) \end{aligned}$$

where ${\tilde{\boldsymbol{W}}}_{\mathrm{HD}}$ could be written as

$$\begin{aligned} {\tilde{\boldsymbol{W}}}_{\mathrm{HD}}=\left[ {\tilde{\boldsymbol{w}}}_{\mathrm{HD}}\boldsymbol{R}{\tilde{\boldsymbol{w}}}_{\mathrm{HD}} \boldsymbol{R}^{2}{\tilde{\boldsymbol{w}}}_{\mathrm{HD}}\boldsymbol{\cdots}\boldsymbol{R}^{N_{\mathrm{HD}}-1}{\tilde{\boldsymbol{w}}}_{\mathrm{HD}} \right],\#\left( \mathrm{AUTONUM} \right) \end{aligned}$$

for a rotational independence. Here ${\tilde{\boldsymbol{w}}}_{\mathrm{HD}}$ is a $N_{\mathrm{HD}}$-length column vector for the connections between two HD neurons with a specific difference of their preferred directions, $\boldsymbol{R}$ is an $N_{\mathrm{HD}}\times N_{\mathrm{HD}}$ rotation matrix

$$\begin{aligned} \boldsymbol{R}=\left[ \begin{matrix} \boldsymbol{0} & 1 \\ \boldsymbol{I}_{N_{\mathrm{HD}}-1} & \boldsymbol{0} \end{matrix} \right],\#\left( \mathrm{AUTONUM} \right) \end{aligned}$$

and $\boldsymbol{I}_{N_{\mathrm{HD}}-1}$ is an $\left( N_{\mathrm{HD}}-1 \right)\times\left( N_{\mathrm{HD}}-1 \right)$ identity matrix. Therefore, $\tilde{\boldsymbol{a}}$ could be written as a circular convolution of two vectors

$$\begin{aligned} \tilde{\boldsymbol{a}}={\tilde{\boldsymbol{w}}}_{\mathrm{HD}}\boldsymbol{*}\tilde{\boldsymbol{f}}.\#\left( \mathrm{AUTONUM} \right) \end{aligned}$$

With the help of Fourier transformation, we give the approximate solution for ${\tilde{\boldsymbol{w}}}_{\mathrm{HD}}$ by estimating its Fourier coefficient vector (see [1] for more details without an analytical solution but rather an approximate solution)

$$\begin{aligned} {\hat{\boldsymbol{w}}}_{\mathrm{HD}}=\frac{\hat{\boldsymbol{a}}\boldsymbol{\cdot}\hat{\boldsymbol{f}}}{\hat{\boldsymbol{f}}\boldsymbol{\cdot}\hat{\boldsymbol{f}}\boldsymbol{+}\lambda},\#\left( \mathrm{AUTONUM} \right) \end{aligned}$$

where $\hat{\boldsymbol{a}}$ and $\hat{\boldsymbol{f}}$ are Fourier coefficient vectors of $\tilde{\boldsymbol{a}}$ and $\tilde{\boldsymbol{f}}$**, ‘**$\boldsymbol{\cdot}$**’** is the dot product, and $\lambda$ is the regularisation term on the flatness of the solution. The estimated ${\tilde{\boldsymbol{W}}}_{\mathrm{HD}}$ is then given from Equation 4. We use this estimated solution to pre-wire the self-connection among neurons in the HD attractor.

During head tuning, the self-connection $\boldsymbol{W}_{\mathrm{HD}}\left( t \right)$ (or the equivalent $\boldsymbol{w}_{\mathrm{HD}}\left( t \right)$) is not updated by synaptic plasticity but rather regulated by the angular velocity $v(t)$ of the head movement. For simplicity, we do not consider anticipatory time interval controlled by angular acceleration [3,4], as we summarize the dorsal tegmental nucleus of Gudden, lateral mammillary nucleus, anterodorsal thalamic nucleus, and dorsal presubiculum in a single structure (our HD attractor). More specifically,

$$\begin{aligned} \boldsymbol{w}_{\mathrm{HD}}\left( t \right)={\tilde{\boldsymbol{w}}}_{\mathrm{HD}}\boldsymbol{-}\tau_{\mathrm{HD}}v\left( t \right)\frac{d{\tilde{\boldsymbol{w}}}_{\mathrm{HD}}}{d\theta},\#\left( \mathrm{AUTONUM} \right) \end{aligned}$$

where $\tau_{\mathrm{HD}}$ is the time constant of HD attractor, and the angular derivative of ${\tilde{\boldsymbol{w}}}_{\mathrm{HD}}\boldsymbol{=}\left\{ \tilde{w}_{l}\boldsymbol{|}1\leq l\leq N_{\mathrm{HD}} \right\}$ is estimated via the central difference between adjacent connections

$$\begin{aligned} \frac{d\tilde{w}_{l}}{d\theta}\boldsymbol{=}\frac{\tilde{w}_{l+1}\boldsymbol{-}\tilde{w}_{l-1}}{2\Delta\theta},\#\left( \mathrm{AUTONUM} \right) \end{aligned}$$

with $\tilde{w}_{N_{\mathrm{HD}}+1}\boldsymbol{=}\tilde{w}_{1}$ and $\tilde{w}_{0}\boldsymbol{=}\tilde{w}_{N_{\mathrm{HD}}}$ for the circularity. We then obtain the self-connection $\boldsymbol{W}_{\mathrm{HD}}\left( t \right)$ for every moment via Equation 4. See [1] for more details on how Equation 8 would keep a unimodal HD signal stable.

The HD signal may drift from the true HD states due to a cumulative path integration error during the head tuning [1]. Therefore, the HD attractor must receive an external signal from dRSC containing landmark information for correction. In this way, the neural dynamics on the HD attractor could be written as

$$\begin{aligned} \tau_{\mathrm{HD}}\frac{d\boldsymbol{a}_{\mathrm{HD}}}{dt}=-\boldsymbol{a}_{\mathrm{HD}}+\boldsymbol{W}_{\mathrm{HD}}\boldsymbol{f}_{\mathrm{HD}}\boldsymbol{+}\boldsymbol{U}_{dRSC2HD} ,\#\left( \mathrm{AUTONUM} \right) \end{aligned}$$

in which a feedforward projection from dRSC transmits the integrated signal $\boldsymbol{f}_{\mathrm{dRSC}}$ to the HD attractor, i.e.

$$\begin{aligned} \boldsymbol{U}_{dRSC2HD}\boldsymbol{=}\frac{g_{dRSC2HD}}{n_{\mathrm{HD}}}\boldsymbol{W}_{dRSC2HD}\boldsymbol{f}_{\mathrm{dRSC}} ,\#\left( \mathrm{AUTONUM} \right) \end{aligned}$$

with the connection containing both 1-to-1 excitation and global inhibition

$$\begin{aligned} \boldsymbol{W}_{dRSC2HD}\boldsymbol{=I}\left( N_{\mathrm{HD}} \right)-\rho_{dRSC2HD}\boldsymbol{I}_{\mathrm{GI}}\left( N_{\mathrm{HD}} \right),\#\left( \mathrm{AUTONUM} \right) \end{aligned}$$

where $N_{\mathrm{dRSC}}=N_{\mathrm{HD}}$ keeping the preference of directions for each cell, and $\rho_{dRSC2HD}$ is the relative strength of inhibitory versus excitatory projection. The 1-to-1 excitatory projection i.e. $\boldsymbol{I}\left( N_{\mathrm{HD}} \right)$ helps the HD attractor to keep the signal not drifting away from the true HD state, whilst global inhibition $\rho_{dRSC2HD}\boldsymbol{I}_{\mathrm{GI}}$ keeps the attractor from saturating when subject to persistent external inputs. Thus, dRSC works as a gateway for visual landmark information to stabilize HD signals against drift.

**Reference**

1. Zhang K. Representation of Spatial Orientation by the Intrinsic Dynamics of the Head-Direction Cell Ensemble: A Theory. J Neurosci. 1996; 16(6):2112–26. doi: 10.1523/JNEUROSCI.5154-08.2009

2. Mardia K V., Jupp PE. Directional Statistics. In John Wiley and Sons Ltd.; 1999. pp. 25–56. doi: 10.1002/9780470316979

3. Blair HT, Sharp PE. Anticipatory head direction signals in anterior thalamus: Evidence for a thalamocortical circuit that integrates angular head motion to compute head direction. J Neurosci. 1995; 15(9):6260–70. doi: 10.1523/jneurosci.15-09-06260.1995

4. Blair HT, Lipscomb BW, Sharp PE. Anticipatory time intervals of head-direction cells in the anterior thalamus of the rat: Implications for path integration in the headdirection circuit. J Neurophysiol. 1997; 78(1):145–59. doi: 10.1152/jn.1997.78.1.145
