# Supplementary material for: A model of head direction and landmark coding in complex environments
Source: PLoS Comput Biol. 2021 Sep 27;17(9):e1009434. doi: 10.1371/journal.pcbi.1009434 (PMC8496825; doi:10.1371/journal.pcbi.1009434)
Supplement: S3 Appendix — (DOCX) [file pcbi.1009434.s003.docx]

**S3 Appendix. Feature-specific visual signals.**

We consider three basic types of signals referring to salient landmarks. The first type is a scaled von Mises distribution with a single peak

$$\begin{aligned} \boldsymbol{f}_{\mathrm{vI}}\left( \varphi, \kappa\right)=\frac{f_{\max}}{e^{\kappa}}e^{\kappa\cos\left( \frac{\pi}{180}\left( \boldsymbol{\theta}-\varphi\right) \right)},\#\left( \mathrm{AUTONUM} \right) \end{aligned}$$

where $\kappa$ is the encoding precision and $\varphi$ is the preferred direction with maximum firing rate $f_{\max}$. This corresponds to inputs used in classical HD modelling studies as a stand-in for cue cards used in experimental environments. The second type (e.g. the broad ‘blue’ cue with low encoding precision $\kappa_{2}$ in Fig 1B) extends $\boldsymbol{f}_{\mathrm{vI}}$ by enlarging the range of high firing rate (via $\psi$), yielding a unimodal signal with less directional specificity

$$\begin{aligned} \boldsymbol{f}_{\mathrm{vII}}\left( \psi, \varphi, \kappa\right)=\int_{-\psi/2}^{\psi/2} \boldsymbol{f}_{\mathrm{vI}}\left( \xi,\kappa\right)d\xi,\#\left( \mathrm{AUTONUM} \right) \end{aligned}$$

which is calculated by the discretized sum with angular sampling gap $\Delta\theta$, as the cumulative distribution function of a von Mises distribution in $\boldsymbol{f}_{\mathrm{vI}}$ could not be expressed as a single analytic function [1]. The third type (e.g. narrow ‘red’ cues with high encoding precision $\kappa_{1}$ in Fig 1B) consists of multiple von Mises distributions with different peaks, yielding a multimodal firing pattern as

$$\begin{aligned} \boldsymbol{f}_{\mathrm{vIII}}\left( \boldsymbol{\Psi}, \kappa\right)=\sum_{\varphi\in\boldsymbol{\Psi}} \boldsymbol{f}_{\mathrm{vI}}\left( \kappa,\varphi\right),\#\left( \mathrm{AUTONUM} \right) \end{aligned}$$

where $\boldsymbol{\Psi}$ is a set of non-overlapping preferred directions so that $\boldsymbol{f}_{\mathrm{vIII}}\left( \boldsymbol{\Psi}, \kappa\right)$ has $\left| \boldsymbol{\Psi} \right|$ peaks.

Visual signals for every feature (including the background noise in S6A Fig), if not zero for all directions, are scaled to the same mean total firing rate, yielding the fluctuation of firing rates about the mean stimulus intensity, inspired by neural adaptation in sensory systems [2]. In addition, this may imply a sense of firing probability over input units encoded as preferred directions. As the scaling assumes the equal distribution of firing-rate intensity over all valid features, a differing distribution among features could be regarded as a result of an external attentional control beyond the HD system. During simulations, we first choose one of the feature-specific visual signals (usually the one with the narrowest peak), then scale it to the maximum firing rate as 1, and take its mean as the reference for scaling signals of other visual features. In addition, a feature-specific visual signal is simply set to zero vector if the egocentric scenery contains no information of that feature. To avoid parallax effects [3] and focus on landmark processing, all cues are simplified as distal cues.

**Reference**

1. Mardia K V., Jupp PE. Directional Statistics. In John Wiley and Sons Ltd.; 1999. pp. 25–56. doi: 10.1002/9780470316979

2. Harth E, Pertile G. The role of inhibition and adaptation in sensory information processing. Kybernetik. 1972; 10(1):32–7. doi: 10.1007/BF00288781

3. Taube JS, Muller RU, Ranck JBJ. Head-direction cells recorded from the postsubiculum in freely moving rats. II. Effects of environmental manipulations. J Neurosci. 1990; 10(2):436–47. doi: https://doi.org/10.1523/JNEUROSCI.10-02-00420.1990
