# Supplementary material for: A model of head direction and landmark coding in complex environments
Source: PLoS Comput Biol. 2021 Sep 27;17(9):e1009434. doi: 10.1371/journal.pcbi.1009434 (PMC8496825; doi:10.1371/journal.pcbi.1009434)
Supplement: S4 Appendix — (DOCX) [file pcbi.1009434.s004.docx]

**S4 Appendix. Alternative algorithms.**

We compare four popular neural algorithms, combined with three sorts of inhibitory connections (global inhibition and lateral inhibition in S1 Appendix, as well as no self-inhibition), to see whether they could similarly result in a sparse and unimodal representation as aLB cells for complex sensory scenery. Here we only choose firing-rate models for simplicity.

The first rule is classic Hebbian learning, i.e. learning without feedback transmission, especially with lateral inhibition among aLB cells. Similar to the competitive learning algorithm [1], lateral inhibition enables winner-takes-all dynamics and yields a sparse encoding of inputs (compare to location-specific firing patterns of place cells [2]). However, simulation results suggest that classic Hebbian learning with or without lateral inhibition cannot generate a unimodal representation for a complex scenery with conflicting cues (S1 Fig, 1^st^ row).

The second rule we consider is the covariance rule. Here synaptic changes in ratio with the covariance of presynaptic and postsynaptic firing rates, i.e. Hebbian covariance learning [3,4], is used to update weights.

$$\begin{aligned} \Delta\boldsymbol{W}^{\left( j \right)}\left( t \right)=\boldsymbol{W}^{\left( j \right)}\left( t+\Delta t \right)-\boldsymbol{W}^{\left( j \right)}\left( t \right)=\eta_{j}\mathbf{V}\left( \boldsymbol{f}\left( t \right) \right){\mathbf{V}\left( \boldsymbol{f}^{\left( j \right)}\left( t \right) \right)}^{T},\#\left( \mathrm{AUTONUM} \right) \end{aligned}$$

where

$$\begin{aligned} \mathbf{V}\left( \boldsymbol{f}\left( t \right) \right)=\boldsymbol{f}\left( t \right)-\int_{0}^{t} \boldsymbol{f}\left( s \right)e^{-\tau_{\mathrm{int}}\left( t-s \right)}ds\#\left( \mathrm{AUTONUM} \right) \end{aligned}$$

stands for the vector of the variation of the firing-rate vector $\boldsymbol{f}\left( t \right)$ from its mean (or expectation) over previous time. Notice that the variation vector $\mathbf{V}$ could be realised in a neuron-like way: Suggesting an interneuron for each input (or output) neuron with a long decaying time $\tau_{\mathrm{int}}$, which receives $\boldsymbol{f}$ from each neuron and provides presynaptic inhibitory feedback. For simplicity, we only use Equations 1 and 2 for the simulation with Hebbian covariance learning.

Compared to the classic Hebbian learning, Hebbian covariance learning with a positive learning rate $\eta_{j}>0$ yields the long-term increases in synaptic connection (i.e. long-term potentiation) when presynaptic and postsynaptic firing rates are positively correlated, long-term decreases in the synaptic connection (i.e. long-term depression) when presynaptic and postsynaptic firing rates are negatively correlated, and a constant synaptic connection when presynaptic and postsynaptic firing rates are uncorrelated. Hebbian covariance learning has previously been suggested to explain associative learning in the hippocampus [5] and primary visual cortex (V1) [6]. However, simulation results with this learning algorithm, lacking a feedback mechanism, fail to yield a unimodal representation with a complex scenery containing conflicting cues (S1 Fig, 2^nd^ row).

For the third rule, we consider synaptic changes with a postsynaptic sliding threshold, i.e. Intrator’s BCM algorithm which maximizes the sparseness of output firing pattern and ensures stability [7],

$$\begin{aligned} \Delta\boldsymbol{W}^{\left( j \right)}=\eta_{j}\left( \boldsymbol{f}\cdot\left( \boldsymbol{f}-\theta_{\boldsymbol{f}} \right)\cdot\frac{d}{d\boldsymbol{x}}\sigma\left( \boldsymbol{f} \right) \right){\boldsymbol{f}^{\left( j \right)}}^{T},\#\left( \mathrm{AUTONUM} \right) \end{aligned}$$

where the sliding threshold

$$\begin{aligned} \theta_{\boldsymbol{f}}\left( t \right)=\int_{0}^{t} \boldsymbol{f}^{2}\left( s \right)e^{-\tau_{\mathrm{int}}\left( t-s \right)}ds.\#\left( \mathrm{AUTONUM} \right) \end{aligned}$$

Intrator’s BCM with $\eta_{j}>0$ yields long-term potentiation when postsynaptic firing rates are above the sliding threshold, long-term depression when postsynaptic firing rates are below the sliding threshold, and constant weights when postsynaptic firing rates reach the sliding threshold. It has been suggested to explain the synaptic changes between the lateral geniculate nucleus and its downstream target V1 [7,8]. Simulation results again show a failure to yield a unimodal representation within a complex scenery (S1 Fig, 3^rd^ row).

For the fourth rule, we consider the original Oja’s Subspace Algorithm (OSA), ensuring the global convergence to a subspace of the span of eigenvectors of the input autocorrelation matrix [9,10]. For comparison we here enable negative weights, as the non-negative weights restriction is only for biological plausibility. Without any self-inhibition, OSA has the same function with principal component analysis and behaves well in visual classification [9]. It was also recently suggested as an explanation for the interaction between place cells and grid cells in the entorhinal-hippocampal network with non-negative synaptic weights [11].

While OSA without lateral inhibition tends to find eigenvectors of the input autocorrelation matrix [9,12], it fails to produce a unimodal representation when the firing pattern of a conflicting cue has a high two-norm over all directions (S1 Fig, 4^th^ row and 1^st^/2^nd^ column; two-norm is 0.2513 for the conflicting ‘red’ cue, compared to 0.1235 for the unambiguous ‘blue’ cue in Fig 2). On the other hand, OSA with lateral inhibition could produce a unimodal representation (S1 Fig, 4^th^ row and 3^rd^ column). These results are in line with the explanation of how the feedback term in OSA with lateral inhibition enables the formation of a sparse and unimodal abstract encoding by supporting winner-takes-all dynamics among aLB cells to permit each aLB cells to learn a unimodal tuning.

In summary, only the OSA with lateral inhibition yields a unimodal representation of landmark bearing (S1 Fig, 4^th^ row), in which the unimodal representation of aLB cells stably emerges at an early stage of learning (S2 Fig).

**Reference**

1. Rumelhart DE, Zipser D. Feature discovery by competitive learning. Cogn Sci. 1985; 9(1):75–112. doi: 10.1016/S0364-0213(85)80010-0

2. Sharp PE. Computer simulation of hippocampal place cells. Psychobiology. 1991; 19(2):103–15. doi: 10.3758/BF03327179

3. Young DL, Poon C-S. Hebbian Covariance Learning. In Springer, Boston, MA; 1998. pp. 73–83. doi: 10.1007/978-1-4757-9077-1_14

4. Sejnowski TJ. Storing covariance with nonlinearly interacting neurons. J Math Biol. 1977; 4(4):303–21. doi: doi.org/10.1007/BF00275079

5. Stanton PK, Sejnowski TJ. Associative long-term depression in the hippocampus induced by hebbian covariance. Nature. 1989; 339(6221):215–8. doi: 10.1038/339215a0

6. Frégnac Y, Shulz D, Thorpe S, Bienenstock E. A cellular analogue of visual cortical plasticity. Nature. 1988; 333(6171):367–70. doi: 10.1038/333367a0

7. Intrator N, Cooper LN. Objective function formulation of the BCM theory of visual cortical plasticity: Statistical connections, stability conditions. Neural Networks. 1992; 5(1):3–17. doi: 10.1016/S0893-6080(05)80003-6

8. Kirkwood A, Rioult MG, Bear MF. Experience-dependent modification of synaptic plasticity in visual cortex. Nature. 1996; 381(6582):526–8. doi: 10.1038/381526a0

9. Oja E. NEURAL NETWORKS, PRINCIPAL COMPONENTS, AND SUBSPACES. Int J Neural Syst. 1989; 01(01):61–8. doi: 10.1142/S0129065789000475

10. Chen T, Hua Y, Yan W. Global convergence of Oja’s subspace algorithm for principal component extraction. IEEE Trans Neural Networks. 1998; 9(1):58–67. doi: 10.1109/72.655030

11. Dordek Y, Soudry D, Meir R, Derdikman D. Extracting grid cell characteristics from place cell inputs using non-negative principal component analysis. Elife. 2016; 5:e10094. doi: 10.7554/elife.10094

12. Oja E. Principal components, minor components, and linear neural networks. Neural Networks. 1992; 5(6):927–35. doi: 10.1016/S0893-6080(05)80089-9
