# Supplementary material for: A model of head direction and landmark coding in complex environments
Source: PLoS Comput Biol. 2021 Sep 27;17(9):e1009434. doi: 10.1371/journal.pcbi.1009434 (PMC8496825; doi:10.1371/journal.pcbi.1009434)
Supplement: S5 Appendix — (DOCX) [file pcbi.1009434.s005.docx]

**S5 Appendix. Simulations with three environments.**

We also test the model with 3 conflicting environments, with scenery in each of them rotated by 120 degrees anticlockwise from the previous one (S7A Fig). The model exhibits the same cellular behaviors as in the two-environmental case in Fig 5 (S7B Fig). This also replicates simulation results from [1], although we employ a richer set of environmental cues constituting complex sceneries. Moreover, the HD representation is stabilized across the whole learning phase (S7C Fig), consistent with the two-environmental case in Fig 6B.

**Reference**

1. Page HJI, Jeffery KJ. Landmark-Based Updating of the Head Direction System by Retrosplenial Cortex: A Computational Model. Front Cell Neurosci. 2018; 12(July):1–17. doi: 10.3389/fncel.2018.00191
