# Supplementary figures and images for: A model of head direction and landmark coding in complex environments
Source: PLoS Comput Biol. 2021 Sep 27;17(9):e1009434. doi: 10.1371/journal.pcbi.1009434 (PMC8496825; doi:10.1371/journal.pcbi.1009434)

# aLB representations in different algorithms

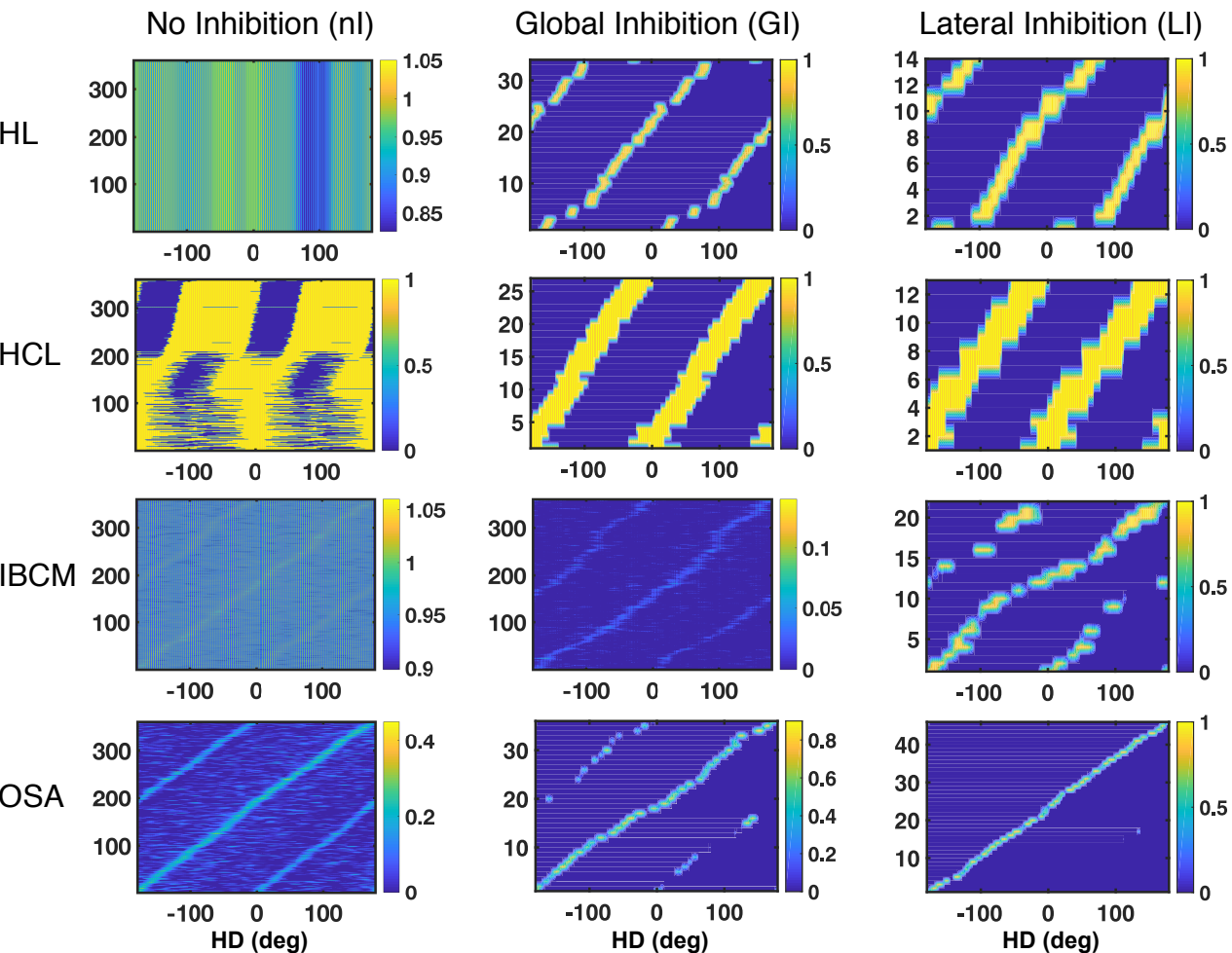

Supplement: S1 Fig — Global representations of aLB cells via alternative algorithms after 20 minutes of learning, following a real rodent’s HD trajectory. Highly activated aLB cells are sorted and labelled by positive numbers on the y-axis. The activity is ordered according to head direction (x-axis). Each row stands for a type of synaptic plasticity, while each column stands for a type of self-inhibition. Note the plot of IBCM-GI shows all aLB cells as none of them have a maximum firing rate above εaLB = 0.5, whilst for other plots the number of recruited aLB cells (i.e. those with firing rate above εaLB) varies among different algorithms (range of y-axis). Only OSA with lateral inhibition (OSA-LI) yields unimodal tuning curves for every recruited aLB cells. Warmer colors represent higher firing rates. Abbreviations: HL: (classic) Hebbian learning; HCL: Hebbian covariance learning; IBCM: Intrator’s BCM; OSA: (original) Oja’s Subspace Algorithm; nI: no self-inhibition; GI: global self-inhibition; LI: lateral self-inhibition; HD: head direction. Notice that OSA-nI and OSA-GI are the OSA with LI replaced by nI or GI, and mOSA is referred to as OSA-LI with non-negative weights restriction. (PDF) [file pcbi.1009434.s006.pdf]

**A**

Weights matrices

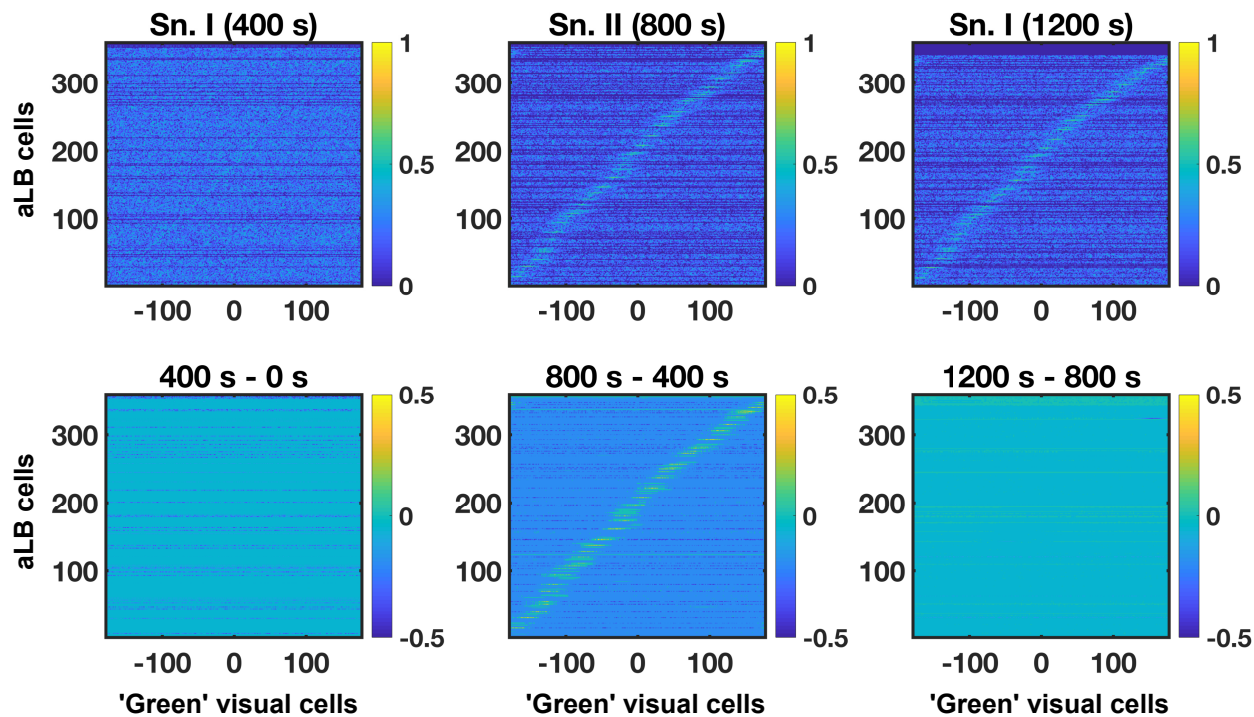**B**

Weights convergence

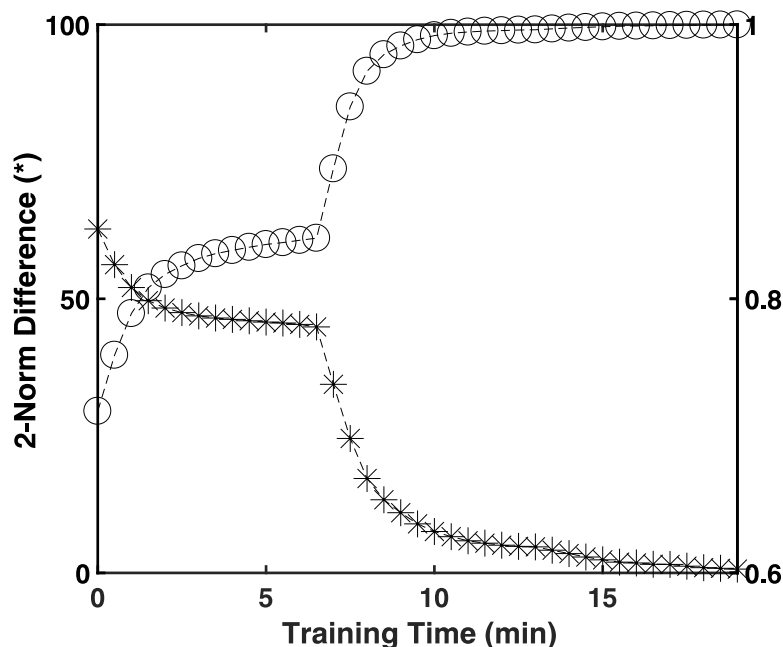**C**

Testing

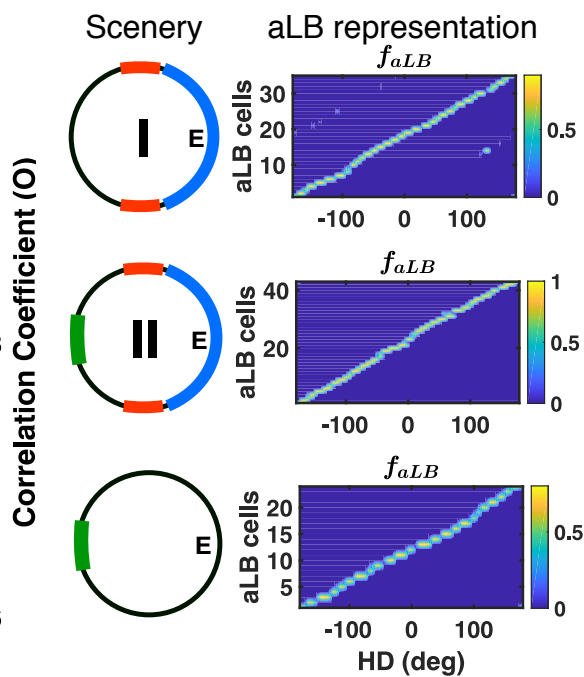

Supplement: S3 Fig — (A) The progress of synaptic weights between ‘green’ visual cells and aLB cells (top), with snapshots taken at the 400 s (left), 800 s (middle), and 1200 s (right), as well as the difference of these weights (bottom). The weights do not show many changes from 800 s to 1200 s, yielding the robustness of novel cue corporation against cue removal. (B) The convergence of synaptic weights during learning. See Fig 2C for illustrations. (C) When the agent just finishes the exposure to Sc. II at 800 s, the global representation of aLB cells are tested on the ‘red-blue’ scenery (top), the ‘red-blue-green’ scenery (middle), and the ‘green’ scenery (bottom). These are nearly the same as the case after the whole learning in Fig 3D (i.e. tested at 1200 s), of which the details are further given in S4 Fig, yielding the robustness of aLB cells encoding against cue removal and previous scene blocking. Warmer colors represent higher firing rates of aLB cells. Abbreviations: Sc.: Scenery; HD: head direction; aLB: abstract landmark bearing; f: firing rate. (PDF) [file pcbi.1009434.s008.pdf]

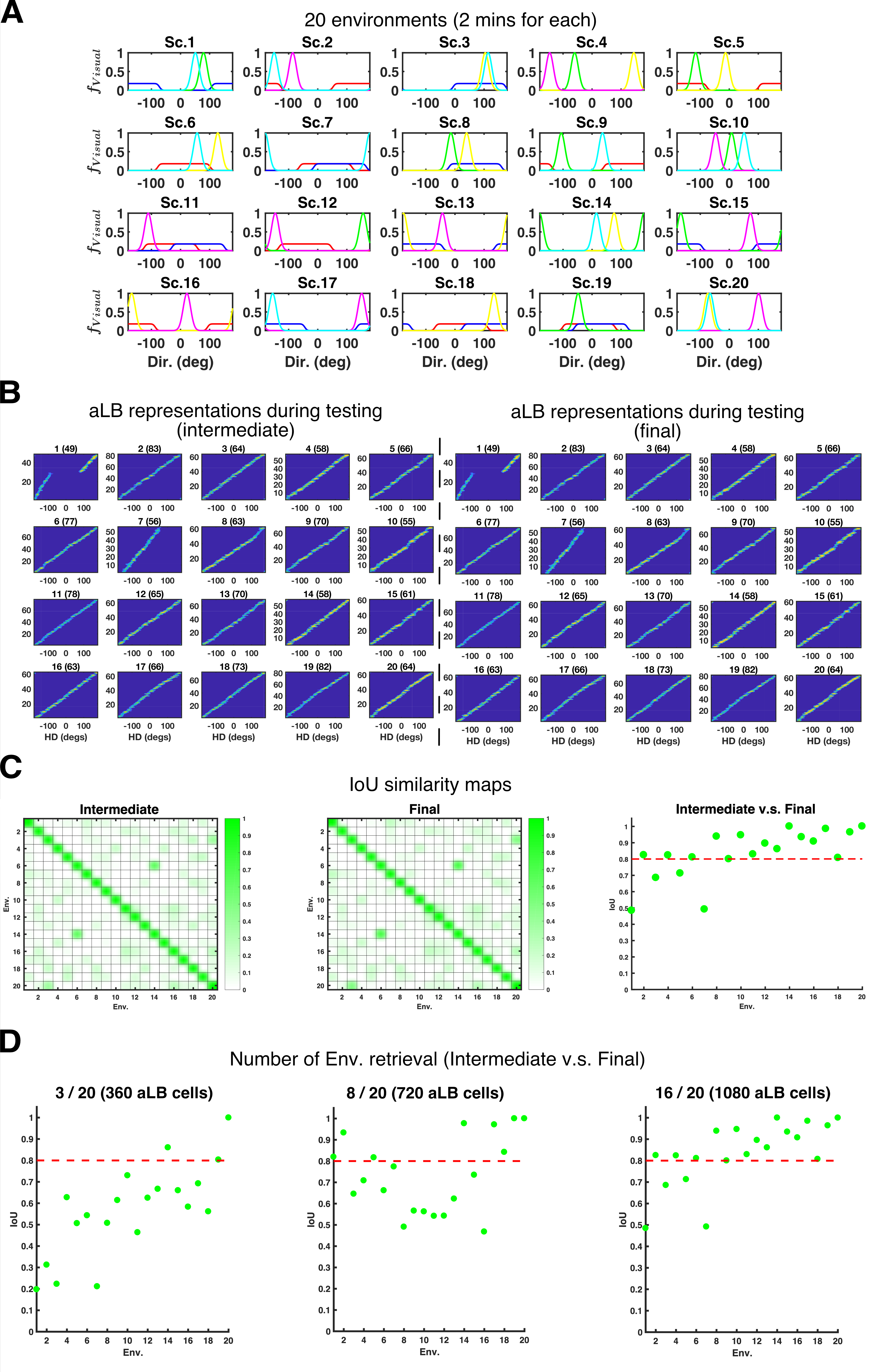

Supplement: S9 Fig — (A) The agent is exposed to 20 environments sequentially with feature-specific visual input signals. In each environment, 3 randomly localized visual cues are randomly selected from 6 cues with independent features (specified in different colors). The learning duration is 2 minutes in each environment, thus 40 minutes in total, of which the HD trajectory is concatenated by two 20-minute HD trajectories used in other simulations (S1 Dataset; also see Fig 1C). (B) Global representations of aLB cells with local weights tested on corresponding sceneries. Titles for each plot refer to the corresponding environment in (A), along with the total number of highly activated aLB cells in brackets. The ’intermediate’ plots provide aLB cell activity based on weights after the first exposure in each individual environment. The ‘final’ plots provide aLB cell activity based on weights after learning in all 20 environments is complete. See Fig 2D for illustrations. Warmer colors represent higher firing rates. (C) IoU similarity maps measuring the similarity of two sets of aLB cell firing patterns when tested on specific sceneries. Axes refer to environments, with 1 as the earliest. The left column provides IoU maps based on ‘intermediate’ weights. The middle column provides IoU map based on ‘final’ weights. The right column provides the IoU index (y-axis) between the first exposure and the end of learning tested on each scenery (x-axis). Green colors represent higher IoU, of which the maximum is 1. Red dashed line refers to IoU as 0.8, with 16 retrieved environments tested above it. (D) Positive correlation between the number of aLB cells and number of retrieved environments. Models are respectively trained with the number of aLB cells as 360 (left), 720 (middle), and 1080 (right, same as S9C right). Number of retrieved environments, which refers to as tested IoU above 0.8 (same as S9C), are stated in each plot title. Abbreviations: HD: head direction; Env.: Environment; Ego.: Egoce [file pcbi.1009434.s014.tiff]

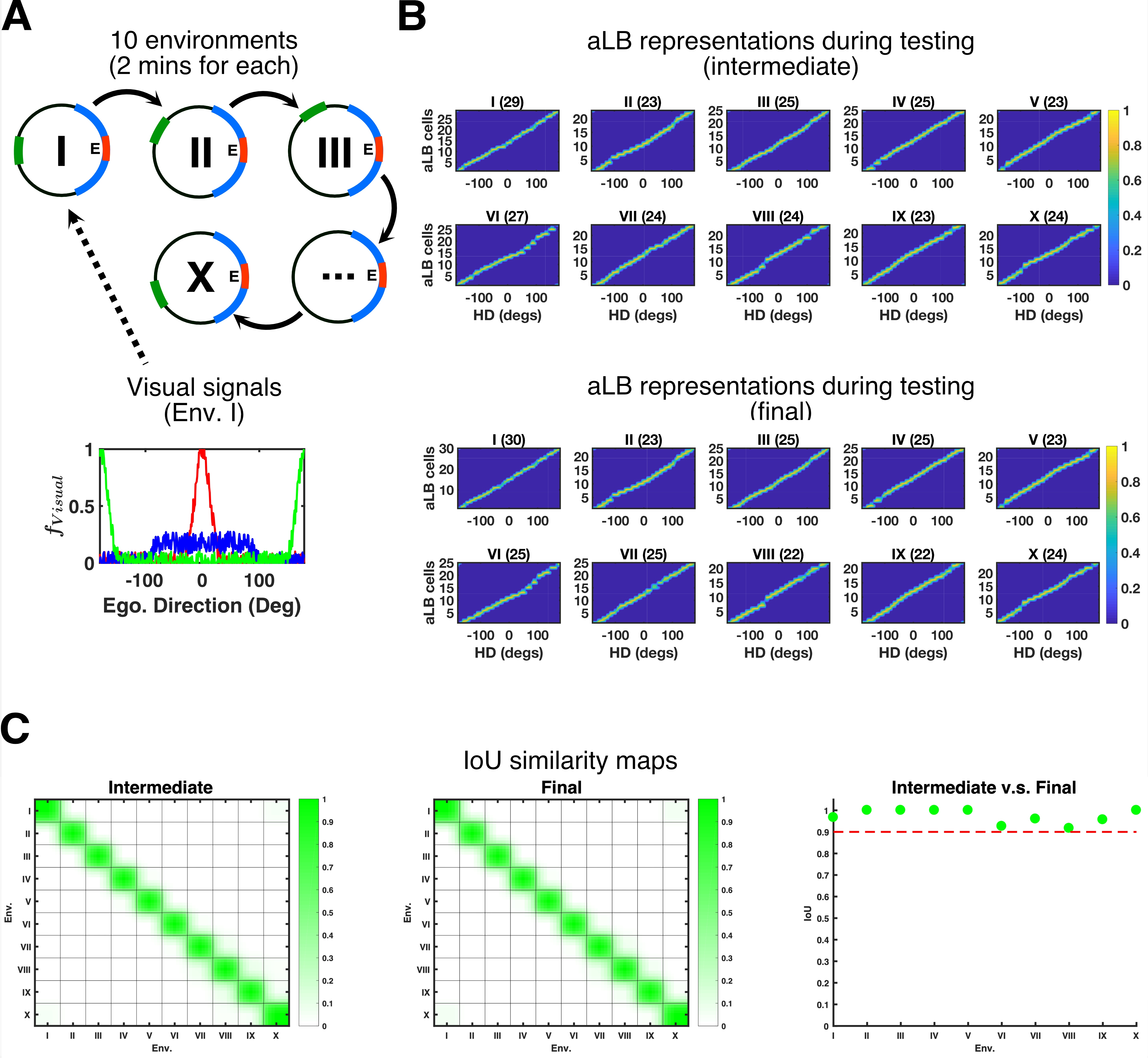

Supplement: S10 Fig — (A-C) Same as Fig 4, except each visual input signal contains uniformly random noise (real-time change every time), with the intensity (i.e. 1-norm across all directions) as 5% of the original visual signal intensity. At least 90% of the recruited aLB cells are preserved over all 10 environments (S10C, right), suggesting the high storage capacity of these aLB cells for long-time scenery retrieval. See corresponding captions in Fig 4 for details. (TIFF) [file pcbi.1009434.s015.tiff]

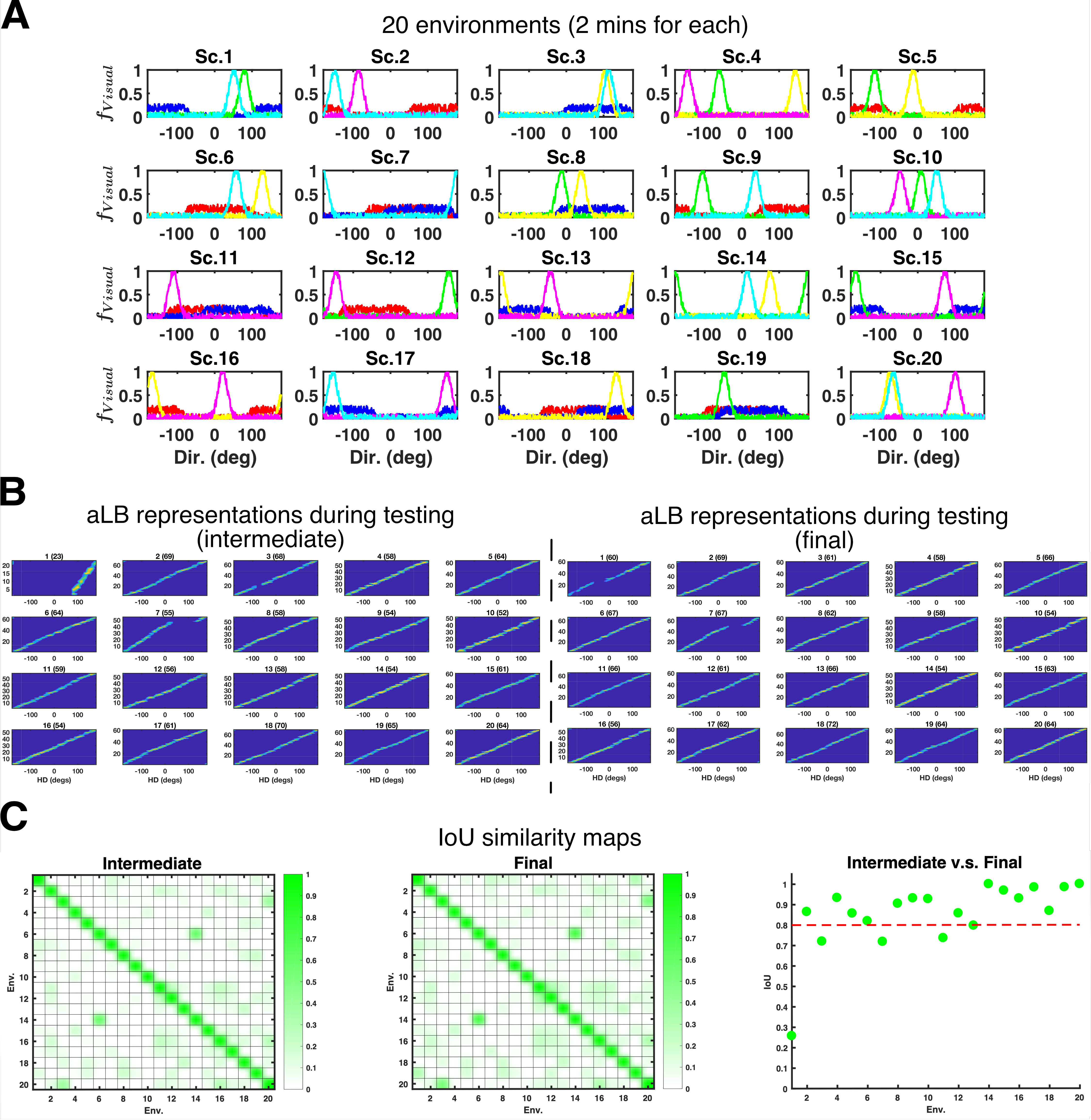

Supplement: S11 Fig — (A-C) Same as S9 Fig, except each visual input signal contains uniformly random noise (real-time change every time), with the intensity as 5% of the original visual signal intensity. At least 80% of the recruited aLB cells are preserved over 16 out of 20 environments (S11C, right), suggesting the high storage capacity of these aLB cells for long-term scenery retrieval. See corresponding captions in S9 Fig for details. (TIFF) [file pcbi.1009434.s016.tiff]

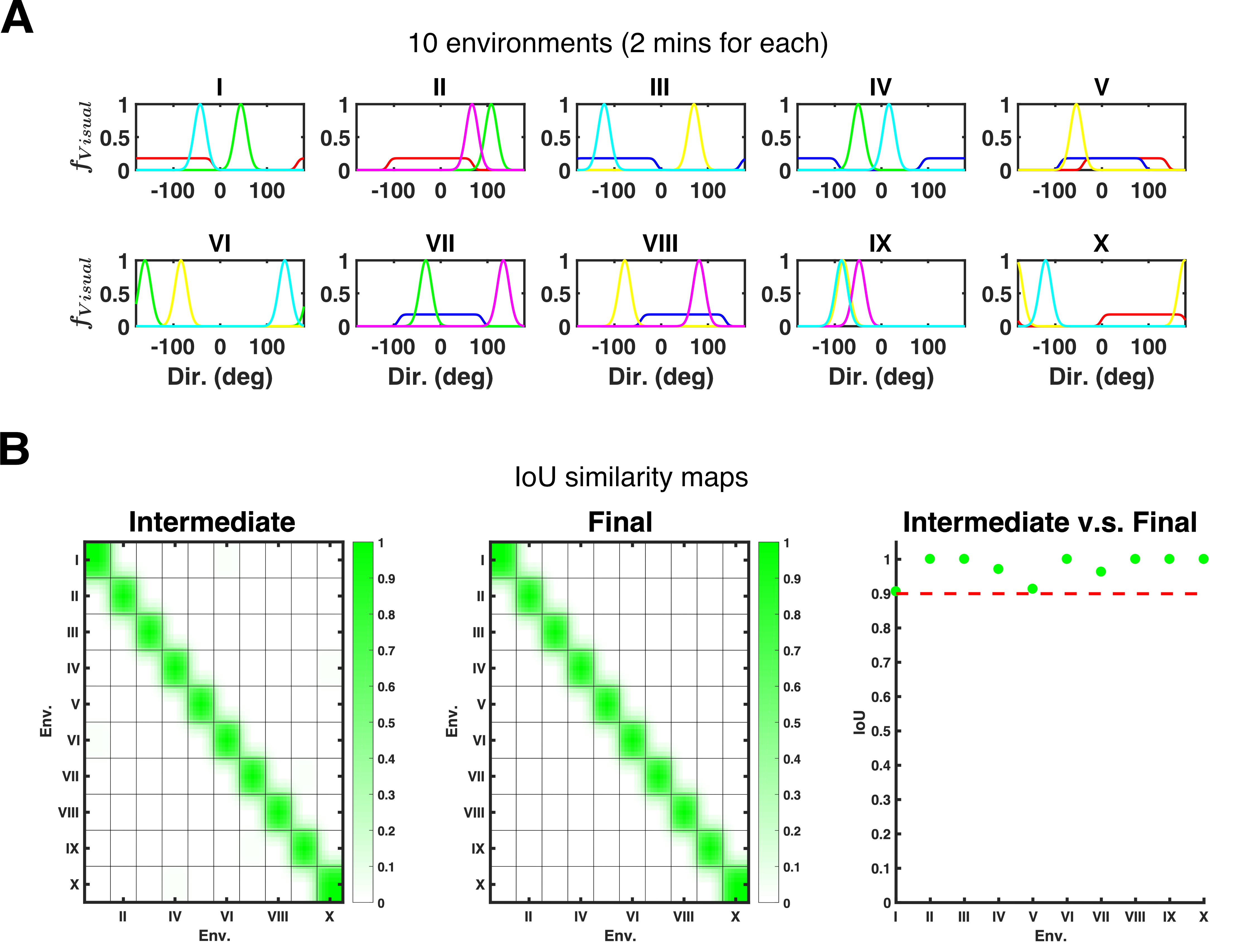

Supplement: S12 Fig — (A) The agent is exposed to 10 environments sequentially with feature-specific visual input signals, with 2 minutes for learning each of them (same as Fig 4A). In each environment, 3 randomly localized visual cues are randomly selected from 6 cues with independent features (specified in different colors), thus richer than Fig 4A. (B) IoU similarity maps measuring the similarity of two sets of aLB cell firing patterns when tested on specific sceneries. At least 90% of the recruited aLB cells are preserved over all 10 environments (S12B, right), suggesting the high storage capacity of these aLB cells for long-term scenery retrieval even with more diverse environmental settings (cf. Fig 4C). See corresponding captions in S9 Fig for details. (TIFF) [file pcbi.1009434.s017.tiff]
