# Supplementary material for: A model of head direction and landmark coding in complex environments
Source: PLoS Comput Biol. 2021 Sep 27;17(9):e1009434. doi: 10.1371/journal.pcbi.1009434 (PMC8496825; doi:10.1371/journal.pcbi.1009434)
Supplement: S6 Fig — (A) A pair of environments with conflicting complex sceneries (top, same as Fig 5A) and V1 signals for each visual feature (bottom, with different colors stand for corresponding features). The low-amplitude grey curve stands for the external background noise derived from a unimodal distribution over all directions. (B) Global representations of aLB and dRSC cells in S6A, tested on the scenery only in Env. I (top; same as Fig 5C, bottom) and the scenery only in Env, II (bottom), both showing bimodal firing patterns as WC-BD cells. See the caption of Fig 5 for more details. (C) A pair of environments with conflicting simple sceneries containing odors (‘blue’ and ‘purple’, independent to each other; top). Only visual signals in the second environment are shown, with odor encoding signals as uniform distributions at all directions, i.e. present for all orientations, for both two environments (bottom, here only show signals in Env. II). (D) Global representations of aLB and dRSC cells in S6C, tested on the scenery only in Env. I (top) and the scenery only in Env. II (bottom). dRSC cells still show bimodal firing patterns when tested in any single environment, suggesting that BC-BD cells may not be solely explained by different odor cues. (E) Global representations of dRSC cells in S6C, tested on each single scenery (Env. I for top and Env, II for bottom) within darkness (i.e. unavailable ‘green’ cue) and with opposite initial HDs (left). The bimodal firing patterns of many dRSC cells are preserved in darkness (right), in accordance with the experimental findings. Here the higher peak of a WC-BC cell combines the HD input from gRSC with residual aLB input from the local odor cue, while the second peak is solely due to gRSC HD inputs (the aLB-odor contribution from the second environment being absent in the first, and vice versa). (F) Global representations of 360 dRSC cells in S6C, of which 180 dRSC cells learn connections slower with aLB cells and gRSC cells than other d [file pcbi.1009434.s011.pdf]

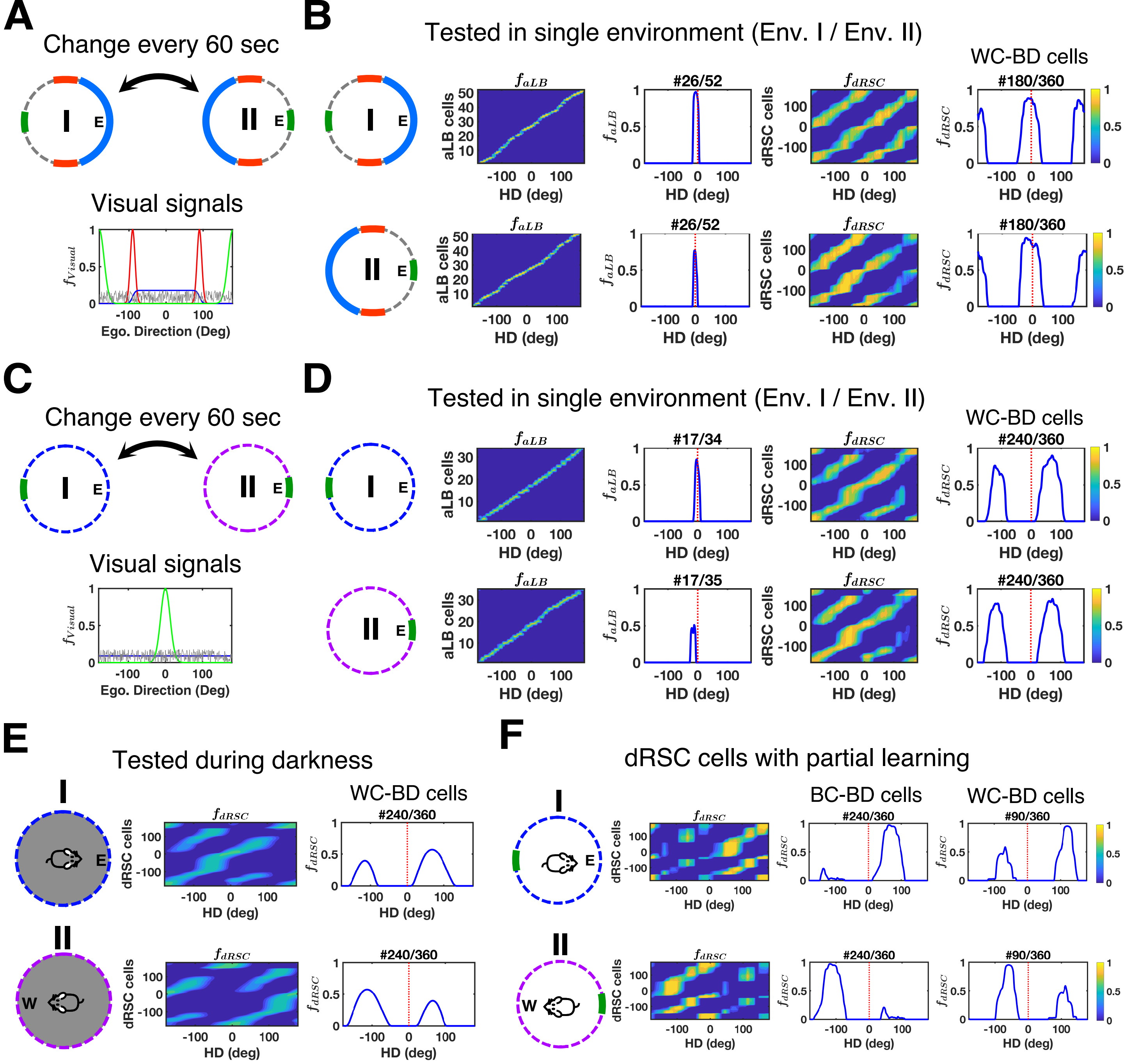
A
Change every 60 sec
I
E
II
E
Visual signals
f_{Visual}
Ego. Direction (Deg)
B
Tested in single environment (Env. I / Env. II)
WC-BD cells
f_{aLB}
aLB cells
#26/52
f_{dRSC}
dRSC cells
#180/360
HD (deg)
C
Change every 60 sec
Visual signals
D
Tested in single environment (Env. I / Env. II)
WC-BD cells
#17/34
#17/35
#240/360
E
Tested during darkness
WC-BD cells
W
F
dRSC cells with partial learning
BC-BD cells
WC-BD cells
#240/360
#90/360
