# Supplementary material for: A model of head direction and landmark coding in complex environments
Source: PLoS Comput Biol. 2021 Sep 27;17(9):e1009434. doi: 10.1371/journal.pcbi.1009434 (PMC8496825; doi:10.1371/journal.pcbi.1009434)
Supplement: S1 Table — Hyperparameters of our two-stage model (with aLB cells) are shown in the third column, and those of the alternative network (without aLB cells) in the fourth column. (DOCX) [file pcbi.1009434.s018.docx]

**S1 Table. Hyperparameters chosen for all simulations in results.**

| **Descriptions** | **Hyperparameters** | **Value** | **Alternative value** |
| --- | --- | --- | --- |
| Time duration | $T_{\mathrm{train}}$ | 1200 s*^S9^ | 1200 s |
|  | $T_{\mathrm{test}}$ | 60 s | 60 s |
| Simulation time step | $\Delta t$ | 0.02 s | 0.02 s |
| Angular sampling gap | $\Delta\theta$ | 1 deg | 1 deg |
| HD encoding precision | $\tilde{\kappa}$ (or $\kappa_{\mathrm{HD}}$) | 15 | 15 |
| V1 encoding precision | $\kappa_{1}$ (narrow) | 80 | 80 |
|  | $\kappa_{2}$ (broad) | 20 | 20 |
|  | $\psi$ (range) | 180 | 180 |
| Parameters in the activation function | $\alpha_{\mathrm{HD}}$ | 20 | 20 |
|  | $\alpha_{\mathrm{aLB}}$ | 0 | n/a |
|  | $\alpha_{\mathrm{dRSC}}$ | 0 | 0 |
|  | $\alpha_{\mathrm{gRSC}}$ | 0 | 0 |
|  | $\beta_{\mathrm{HD}}$ | 0.08 | 0.08 |
|  | $\beta_{\mathrm{aLB}}$ | 0.05 | n/a |
|  | $\beta_{\mathrm{dRSC}}$ | 0.04 | 0.04 |
|  | $\beta_{\mathrm{gRSC}}$ | 0.04 | 0.04 |
| Threshold of the activation level | $\gamma_{\mathrm{HD}}$ | 0 | 0 |
|  | $\gamma_{\mathrm{aLB}}$ | 0 | n/a |
|  | $\gamma_{\mathrm{dRSC}}$ | 0 | 0 |
|  | $\gamma_{\mathrm{gRSC}}$ | 0 | 0 |
| Membrane potential time constant | $\tau_{\mathrm{HD}}$ | 0.02 s | 0.02 s |
|  | $\tau_{\mathrm{aLB}}$ | 0.02 s | n/a |
|  | $\tau_{\mathrm{dRSC}}$ | 0.02 s | 0.02 s |
|  | $\tau_{\mathrm{gRSC}}$ | 0.02 s | 0.02 s |
|  | $\tau_{\mathrm{int}}$ | 1 s^+^ | n/a |
| Normalized number of neurons  (Total number of neurons $N_{j}=360n_{j}$) | $n_{\mathrm{Vis}}$ (each feature) | 1 | 1 |
|  | $n_{\mathrm{HD}}$ | 1 | 1 |
|  | $n_{\mathrm{aLB}}$ | 1*^S9^ | n/a |
|  | $n_{\mathrm{dRSC}}$ | 1 | 1 |
|  | $n_{\mathrm{gRSC}}$ | 1 | 1 |
| Gain factor | $g_{V2aLB}$ | 2*^S9^ | n/a |
|  | $g_{\mathrm{aLB}}$ | $-$500 | n/a |
|  | $g_{aLB2dRSC}$ | 50 | n/a |
|  | $g_{V2dRSC}$ | n/a | 3 |
|  | $g_{gRSC2dRSC}$ | 5 | 3 |
|  | $g_{\mathrm{dRSC}}$ | $-$50 | $-$100 |
|  | $g_{\mathrm{gRSC}}$ | $-$50 | $-$50 |
|  | $g_{dRSC2HD}$ | $0.1$ | $0.1$ |
|  | $\rho_{dRSC2HD}$ | 2 | 2 |
|  | $g_{HD2gRSC}$ | 50 | 50 |
| Maximum weights connection | $w_{V2aLB}$ | 10^+^ | n/a |
|  | $w_{aLB2dRSC}$ | 1 | n/a |
|  | $w_{V2dRSC}$ | n/a | $N_{F}$ |
|  | $w_{gRSC2dRSC}$ | 1 | 1 |
|  | $w_{dRSC2HD}$ | $1$ | $1$ |
| Learning rate  (based on $\Delta t=0.02$ s) | $\eta_{V2aLB}$ | 1e$-$3*^S9^ | n/a |
|  | $\eta_{aLB2dRSC}$ | 1e$-$4*^S6F^ | n/a |
|  | $\eta_{V2dRSC}$ | n/a | 1e$-$4 |
|  | $\eta_{gRSC2dRSC}$ | 5e$-$5*^S6F^ | 5e$-$5 |
| Expected maximum firing rate | $f_{\max}$ (visual) | 1 | 1 |
|  | $\tilde{f}_{\max}$ (HD attractor) | 0.8 | 0.8 |
| Regularization term | $\lambda$ (HD attractor) | 1 | 1 |
| Activity threshold | $\varepsilon_{\mathrm{aLB}}$ | 0.5*^S9^ | 0.5 |

Hyperparameters of our two-stage model (with aLB cells) are shown in the third column, and those of the alternative network (without aLB cells) in the fourth column.

* The value may change in specific simulations mentioned in the context.

*S9 The value is changed for S9 Fig as the following: $T_{\mathrm{train}}=2400$ s, $n_{\mathrm{aLB}}=3$, $g_{V2aLB}=10$, $\eta_{V2aLB}=1e-4$, $\varepsilon_{\mathrm{aLB}}=0.3$ .

*S6F For 180 dRSC cells, the value is changed for S6F Fig as the following: $\eta_{aLB2dRSC}=1e-7$, $\eta_{gRSC2dRSC}=5e-8$,

whilst for the rest of dRSC cells having the same value mentioned in the table.

^+^ The value is only used in alternative algorithms with aLB cells.

n/a: Not available due to the structure of the model or framework.

$N_{F}$: Number of utilized features.
