# Supplementary material for: A model of head direction and landmark coding in complex environments
Source: PLoS Comput Biol. 2021 Sep 27;17(9):e1009434. doi: 10.1371/journal.pcbi.1009434 (PMC8496825; doi:10.1371/journal.pcbi.1009434)
Supplement: S2 Table — (DOCX) [file pcbi.1009434.s019.docx]

**S2 Table. Synaptic connections in the model.**

| **Connection** | **Algorithm** |
| --- | --- |
| $W_{Vis2aLB}$* | Modified Oja’s Subspace Algorithm^+^ |
| $W_{\mathrm{aLB}}$* | Lateral self-inhibition^+^ |
| $W_{aLB2dRSC}$* | Classic Hebbian Learning |
| $W_{Vis2dRSC}$** | Classic Hebbian Learning |
| $W_{gRSC2dRSC}$ | Classic Hebbian Learning |
| $W_{\mathrm{dRSC}}$ | Global self-inhibition |
| $W_{\mathrm{gRSC}}$ | Global self-inhibition |
| $W_{dRSC2HD}$ | 1-to-1 feedforward & Global inhibition |
| $W_{HD2gRSC}$ | 1-to-1 feedforward |
| $W_{\mathrm{HD}}$ | Pre-wired and controlled by HD velocity |

* The connection only occurs in our two-stage model with aLB cells.

** The connection only occurs in the alternative network without aLB cells, i.e. with a direct projection from V1 to dRSC.

^+^ The mechanism of synaptic plasticity varies under alternative algorithms.
