# Supplementary material for: A model of head direction and landmark coding in complex environments
Source: PLoS Comput Biol. 2021 Sep 27;17(9):e1009434. doi: 10.1371/journal.pcbi.1009434 (PMC8496825; doi:10.1371/journal.pcbi.1009434)
Supplement: Attachment — Submitted filename: Review-PLOSCompBio_HDmodel.pdf [file pcbi.1009434.s021.pdf]

The authors present a model of head direction cells that accounts for the differences in connectivity and believed functionality between the dRSC and gRSC subregions. Their model makes use of a novel type of cell called abstract landmark bearing cells that encode visual scenes through unimodal responses dependent on the animal's egocentric facing direction. They show these cells give the system desirable properties such as a large capacity and robustness to unstable cues, and they provide a means for stabilizing HD activity using more complex visual scenes than are typically considered in models.

I recommend this article for publication with minor revisions (see below). The paper is well-written, and the authors present several simulations of various situations to test the model and compare it to related models. The model leads to testable predictions and interesting biological implications.

Specific Comments

1. The authors show in Fig. 4 that the system can maintain unique representations of 10 distinct environments. However, the environments used are very similar, differing only by the angle of one cue. This is a solid test for pattern separation, but I wonder whether the consistency of most cues in each environment contributes to the stability of the representation of each (w/o being erased due to learning a new environment). The authors should address this question.
2. The authors compare the aLB cells to hippocampal place cells (most notably on pg. 40-41). This is quite interesting. It is generally theorized that the sparse, strong inputs from the dentate gyrus lead to the pattern separation and capacity of place cells. Here, the authors achieve sparsity through lateral inhibition (L375). Since the authors discuss on pg. 17 how the modified OSA learning rule is necessary for the model to be effective, they should also discuss whether afferents from cells with firing properties such as those in DG may allow other plasticity rules to be used here.
3. L302: Give a brief description of the idea of OSA so the reader can know the essence of the learning rule without having to refer to citations. You give the exact form of OSA in L372, but it would be helpful to include a sentence describing it earlier.
4. L90: the reference to Table 1 for abbreviations seems randomly placed in the middle of this paragraph. It would make more sense to reference Table 1 after the first abbreviation.
5. What do the white lines in Fig. 2D represent? These are not mentioned in the caption. The vertical line appears again in some plots in Fig. 3B and Fig. 6B.
6. Experiment of Fig. 4: This is an interesting experiment, but it should be explained better. Specifically:
    a. The caption for B should explain "first" and "last," which is not explained until C.
    b. I find the words "first" and "last" confusing. Based on the description on pg. 26, the agent experiences each environment only once, so it's odd to refer to a first exposure vs. a last exposure to a specific environment. Furthermore, "first exposure" typically means when the animal first enters an environment, but the authors use this term to refer to the time after learning the environment. Terms such as "intermediate" and "final" would be more clear.
    c. In the plots in C, based on my understanding, there should be 100 discrete data points here, one for each pair of environments. The authors should change the axis limits to show the first environment, which appears to be missing. Also, what does the green band represent?
7. Fig 5A: There appears to be a mistake in the schematic (bottom red cue is off track)

Typos

1. L63: "an animals' head" should be "an animal's head"

2. L174: a parenthesis is closed without being opened
3. L191: take out the comma after “inputs”
4. L244: “which *is* in turn” should be “which *are* in turn,” to make clear that you’re referring to the signals
5. L382: “some of which multimodal” should be “some of which *are* multimodal”
6. L728: I believe “thought” should be “throughout”
7. L798: run-on sentence
