# Supplementary material for: A model of head direction and landmark coding in complex environments
Source: PLoS Comput Biol. 2021 Sep 27;17(9):e1009434. doi: 10.1371/journal.pcbi.1009434 (PMC8496825; doi:10.1371/journal.pcbi.1009434)
Supplement: Attachment — Submitted filename: Response_Letter.docx [file pcbi.1009434.s022.docx]

Dear editor,

We thank the editor and the reviewers for their time and comments.

As requested, we submit the manuscript with tracked changes, as well as a separate, clean manuscript version (with all revisions accepted). Below we answer each comment raised in the review.

**Comments from Editor**

*1) Both reviewers mention that the method used to achieve sparse coding may be unrealistic or too extreme. The potential effects on the model of alternative sparse encoding should be considered.*

aLB cells here are regarded as providing a latent representation of the ‘orientation’ of the current egocentric scenery. They need not show the firing pattern akin to head direction cells (i.e. be Gaussian-like), especially due to the effect of the lateral inhibition.

We note that the mechanism (inspired by competitive learning from Rumelhart & Zipser, 1985) is in line with several other published studies with winner-take-all dynamics, e.g. hippocampal place cells (Sharp, 1991; Coultrip et al., 1992), visual object recognition (Riesenhuber & Poggio, 1999), and selective attention (Carpenter & Grossberg, 1987). Moreover, these models simply select the most strongly activated units for learning while ignoring the activity of other co-active cells. We consider this method of generating sparse code in previous models legitimate, especially when focusing on the algorithmic level. Nevertheless, we would like to stress that we have made an extra effort to introduce lateral inhibition as a biologically plausible mechanism, implemented as a neural mechanism in simulation, rather than simply selecting the most active unit and ignoring the rest. As such we do not believe our mechanism to be too extreme. Naturally all models must make simplifications/idealisations, but winner-take-all dynamics are a commonly accepted coding principle with strong experimental support (e.g. Sayer et al., 1990; Zénon et al., 2009; Kim et al., 2020) [complete references are given at the end of this file].

All that said, we do try to outline how less sparse aLB activity may be possible. For instance, when subsets of aLB cells do not share inhibitive connections with each other, they may fire together (meanwhile suppressing other aLB cells with inhibitive connections with any of them). This is now mentioned in the *Lateral inhibition and feedback transmission* subsection of *Discussion* section.

*2) A more thorough justification of claims about the capacity of the network is needed, e.g., how this is affected by noise.*

Since both referees comment on this issue from slightly different angles, we reply to each separately below. In summary, we provide new simulations to flesh out the capacity results, and to distinguish capacity from pattern separation.

*3) Reviewer #2's comment that the 'modified' Oja rule can effectively be reduced to the original rule by rescaling needs to be satisfactorily addressed.*

The modified OSA can indeed be rewritten without $\zeta$ in Equation 7 by rescaling $\eta_{V2aLB}$ and $g_{V2aLB}$ in Equations 6 and 7 (see S1 Table). We have changed this in manuscript. Please also see our reply to Reviewer #2’s first major comment. Note that we still refer to the algorithm as mOSA because of the added lateral inhibition and the non-negativity constraint (compared to the original OSA).

**Comments from Reviewer #1**

*1. The authors show in Fig. 4 that the system can maintain unique representations of 10 distinct environments. However, the environments used are very similar, differing only by the angle of one cue. This is a solid test for pattern separation, but I wonder whether the consistency of most cues in each environment contributes to the stability of the representation of each (w/o being erased due to learning a new environment). The authors should address this question.*

We reasoned that if the system can distinguish very similar environments (as an edge case), then the model can also distinguish and store more varied environments. Here ‘distinguished’ is synonymous with ‘recruiting different sets of aLB cells’ (see Equation 7 of mOSA where no learning would occur with $f_{\mathrm{aLB}}=0$). To re-enforce this claim and address the reviewer’s comment we present extra simulations and add results as supplementary figures (S9 and S11 Figs with 20 environments; S12 Fig with 10 environments directly compared to Fig 4), where each of the environments is populated by a random selection of 3 out of 6 cues with independent features (S9A, S11A, and S12A Figs). That is, we added 3 more input channels to the visual inputs, capable of representing 3 more input features. The resulting environments are more varied, yet capacity is preserved (S9C, S11C, and S12B Figs). We also show the positive correlation between the number of aLB cells and its storage capacity (S9D Fig). Please also see our reply to reviewer2’s 3^rd^ major point. These results should support our claims regarding capacity (as compared to pattern separation).

The issue is also taken up again in the section *Coping with environmental changes, and ‘remapping’* subsection in the *Discussion* section, ‘when the novel cue is more salient than the remaining array of cues, a novel set of aLB cells is recruited.’ (Figs 3C and 4); otherwise ‘the mOSA algorithm can disconnect aLB cells from such ephemeral landmarks via long-term depression.’ (Figs 3A and 3B). The saliency of new cues in the new environment, rather than the consistency of cues across environments, influences whether a novel set of aLB cells is recruited for learning so that the pattern separation is achieved. We have endeavoured to make this clearer throughout the manuscript. We also mention pattern separation in this context now.

*2. The authors compare the aLB cells to hippocampal place cells (most notably on pg. 40-41). This is quite interesting. It is generally theorized that the sparse, strong inputs from the dentate gyrus lead to the pattern separation and capacity of place cells. Here, the authors achieve sparsity through lateral inhibition (L375). Since the authors discuss on pg. 17 how the modified OSA learning rule is necessary for the model to be effective, they should also discuss whether afferents from cells with firing properties such as those in DG may allow other plasticity rules to be used here.*

This is an excellent point and we happily oblige. We have expanded the subsection *Lateral inhibition and feedback transmission* subsection of *Discussion* section to briefly discuss these points.

*3. L302: Give a brief description of the idea of OSA so the reader can know the essence of the learning rule without having to refer to citations. You give the exact form of OSA in L372, but it would be helpful to include a sentence describing it earlier.*

We agree that this will help build intuition for the reader. A brief description of the idea of OSA has now been added to the second paragraph of the *Abstract landmark bearing and the mOSA learning algorithm* section of *Materials and Methods* section.

*4. L90: the reference to Table 1 for abbreviations seems randomly placed in the middle of this paragraph. It would make more sense to reference Table 1 after the first abbreviation.*

Table 1 is now moved to the place below the second paragraph of *Introduction* section.

*5. What do the white lines in Fig. 2D represent? These are not mentioned in the caption. The vertical line appears again in some plots in Fig. 3B and Fig. 6B.*

White lines occurred due to a technical problem in saving EPS images. All figures have been corrected.

*6. Experiment of Fig. 4: This is an interesting experiment, but it should be explained better. Specifically:*

*a. The caption for B should explain “first” and “last,” which is not explained until C.*

*b. I find the words “first” and “last” confusing. Based on the description on pg. 26, the*

*agent experiences each environment only once, so it’s odd to refer to a first exposure vs. a last exposure to a specific environment. Furthermore, “first exposure” typically means when the animal first enters an environment, but the authors use this term to refer to the time after learning the environment. Terms such as “intermediate” and “final” would be more clear.*

*c. In the plots in C, based on my understanding, there should be 100 discrete data points here, one for each pair of environments. The authors should change the axis limits to show the first environment, which appears to be missing. Also, what does the green band represent?*

a. The explanation is now moved to the caption for B.
b. The terminology ‘first’ and ‘last’ is now changed to ‘intermediate’ and ‘final’ respectively in the whole *High capacity of scene-based encoding* subsection of the *Results* section.
c. The intermediate green band occurred as a colour gradient. As using colour gradient here is indeed confusing to recognize a 10-by-10 matrix, we have revised this so that the 10-by-10 discrete data points should be much clearer.

*7. Fig 5A: There appears to be a mistake in the schematic (bottom red cue is off track)*

Revised.

*Typos*

*1. L63: “an animals’ head” should be “an animal’s head”*

*2. L174: a parenthesis is closed without being opened*

*3. L191: take out the comma after “inputs”*

*4. L244: “which is in turn” should be “which are in turn,” to make clear that you’re referring to the signals*

*5. L382: “some of which multimodal” should be “some of which are multimodal”*

*6. L728: I believe “thought” should be “throughout”*

*7. L798: run-on sentence*

These points are now all revised.

We thank Reviewer 1 for his/her time and effort and the constructive review.

**Comments from Reviewer #2**

*## Major points*

*The authors modify the Oja rule on three points (l. 303), the third being a rescaling of the feedback term. It seems the one can always rescale W_{V2aLB} and \eta in eq. 7 so that the learning dynamics is equivalent to a dynamics where \xi =1 as in the original Oja rule (notice that W_V2aLB in equation 6 appears with a prefactor g_V2aLB that can be adjusted accordingly). The modifications that the authors propose on the Oja rule do not seem to be fundamental.*

The reviewer is correct that the modified OSA can indeed be rewritten without $\zeta$ in Equation 7 by rescaling $\eta_{V2aLB}$ and $g_{V2aLB}$in Equations 6 and 7 (see S1 Table). Therefore, we have modified the corresponding parts of the manuscript to reflect this (including Equation 7).

However, we note that the interpretation of what happens at the level of biological implementation changes quite drastically. Rather than upregulating the effect of the feedback term ($\zeta$ in Equation 7b; and the effect of the feedback term could potentially map onto different phases of the theta rhythm, see Discussion section), modulating the scaling factor $g$ suggests effects on the forward connection converging onto aLB cells from visual areas (rather than a back projection). For this reason, we briefly mention the original form of the equation in the manuscript. However, the referee is correct that at the algorithmic level both model produce the same results.

*The learning rule on the aLB population leads to a winner take all dynamics whereby only a single aLB cell is active at any given time. This seems somewhat artificial. I wonder whether it would be possible to couple the aLB cells in a different way to obtain a sparse activity without the extreme sparsity that the authors describe.*

A less sparse version aLB cells is potentially technically feasible. For instance, when multiple aLB cells do not share inhibitive connections with each other (or the connection are more graded), they may fire together (meanwhile suppressing other aLB cells with inhibitive connection with any of them). This could go as far as providing a Gaussian-like output firing pattern (e.g. Equation 8 in S2 Appendix). We agree that there is room to explore this further and note so in the text. Please also see our reply to the editor’s comment above.

*The authors claim that the network has a high capacity (l 594) and that it scales with the number of aLB cells l 991. Could the author make that claim more precise? The capacity was first defined in Hopfield networks, where the capacity scales linearly with the number of neurons. In the present manuscript, the situation is different as thermal noise is not present. I think the author should explore the robustness of their learning to input noise or better define the capacity.*

The capacity here is referred to as the number of distinct environments that can be stored for future HD recall across multiple distinct environments, as mentioned in the *High capacity of scene-based encoding* subsection of the *Results* section. Due to the considerable variation in the number of recruited aLB cells and varying overlap (in recruited cells) between environments, it is hard to estimate the capacity in analytical detail. We also believe that this is an issue separate from the robustness against input noise, which we did examine through Figs 3 (with the saliency issue) and 5 (with input noise for simulation).

**Capacity:** Please note, this question shows some overlap with comment 1 by reviewer #1. Hence we invoke the additional material provided to answer that question here as well. To address the question by Reviewer #1 on pattern separation vs capacity, we added new simulations in S9-S11 Figs, with S9 Fig added to the *Results* section. In S9 Fig we conduct a simulation (similar to Fig 4) with 20 environments that are less similar (please see reply to the first comment of Reviewer #1). At least 80% of the recruited aLB cells are preserved over time for 16 out of 20 environments (S9C Fig, right), suggesting the storage capacity of these aLB cells of 16 out of 20 different visual sceneries (given the minimum retrieval IoU as 0.8). We also show the positive correlation with the number of aLB cells and its storage capacity (S9D Fig), by comparing the capacities obtained with differently sized aLB reservoirs. Please note, going beyond 1080 aLB cells required prohibitively long simulation times for this set of simulations with 20 environments. Hence we compared the original simulations to new simulations with lower total numbers of aLB cells. Given the number of aLB cells in our largest simulation is $O(1000)$, which is a very modest number compared to typical cortical populations, we believe the current model could scale well.

**Noise:** In S10-S11 Figs we also show that the capacity showcased in Fig 4 and S9 Fig is maintained against a certain level of visual input noise (real-time change every time step; noise intensity as 5% of the original visual signal intensity).

*It is observed experimentally that both between compartment bidirectional cells (BC-BD) and within compartment bidirectional (WC-BD) cells fire in the dark. It seems that the recurrence in the present model would account for firing in the dark, but I didn’t find it claimed anywhere. Could the authors comment on that?*

We now provide an additional figure panel in Fig S6E which shows that the odour cues can support WC-BD cells in darkness. Within a given environment (in the testing phase, in darkness), 1 peak of a WC-BC cell combines the HD input from gRSC with residual aLB input from the local odour cue (the higher peak), while the second peak is solely due to gRSC HD inputs (the aLB-odour contribution from the second environment being absent in the first, and vice versa).

*Also, BC-BD cells are not captured by the current model, and the authors suggest in the discussion that they could arise from a slower learning rate. Could the authors consider a heterogeneous learning rate and try to see whether BC-BD cells would appear?*

Trying a heterogenous learning rate is an interesting suggestion. We now show supplementary results (Fig S6F) where the learning rates for incoming connections to a subset of dRSC cells are set to very low levels, in which some cells do indeed show activity akin to BC-BD cells (Fig S6F, middle) whilst others show activity akin to WC-BD cells (Fig S6F, left). Note that decreasing the learning rate globally (i.e. for all dRSC cells) towards such low levels will lead to the re-emergence of drift in the HD attractor, as visual feedback would be not learned fast enough.

We now mention this new result in the results and discussion session. Exploring heterogeneous learning rates in more detail, e.g. the effect of different proportions of cells with different learning rates or the distribution of learning rates, will provide for very interesting follow-up work, and we thank the referee for this suggestion.

*## Minor points*

*Many of the plots are challenging to read. Most of the time, the axes are not labeled, and titles are missing.*

We apologise for any inconvenience. We have checked and revised (where necessary) all figures with all corresponding simulations. We have also checked (re-run) all simulations to confirm they produce the same results with the rewritten mOSA. Besides, the following figures are revised for clarification:

- Fig 2: Axes labelled; Titles added;
- Fig 3: Axes labelled; Titles added; Caption revised;
- Fig 4: Axes labelled; Titles revised; Colorbars added; IoU maps in Fig 4C changed to heatmaps and scatterplots respectively; Caption revised;
- Fig 5: Axes labelled; Titles revised; Colorbars added; Explanation of simulation revised in the caption; Caption revised;
- Fig 6: Axes labelled; Titles revised; Colorbars added; Caption revised;
- Fig 7: Axes labelled; Titles revised; Colorbars added; Caption revised;
- Fig 8: Axes labelled; Titles revised; Colorbars added; Caption revised;
- S1 Fig: Due to the new mOSA without $\zeta$, alternative algorithms are restricted to HL, HCL, IBCM, and original OSA; Axes labelled; Caption revised;
- S2 Fig: Caption revised due to the new simulation; Axes labelled;
- S3 Fig: Axes labelled; Titles added; Caption revised;
- S5 Fig: Titles revised; Caption revised;
- S6 Fig: Axes labelled; Titles revised; Colorbars added; Caption revised; S6E-F Figs and relative caption added; Caption revised;
- S7 Fig: Axes labelled; Titles revised; Colorbars added; Caption revised;
- S8 Fig: Axes labelled; Colorbars added; Caption and drift angles revised;
- S9-S12 Figs: Added.

Please note, some of the plots have no labelled axes for plotting simplicity when they appear in groups of plots. I.e., for groups of plots that share the same axes common x-axes labels are added to the bottom-most plots and common y-axes labels are added to the leftmost plots.

*In fig. 4B, The y-axis and the order of cells should be different (they seem individually reordered). I am curious to see whether there is a correlation on the order between the different environments (even if the Jaccard index is almost zero between environments).*

As the Jaccard index is almost zero between any two different environments (Fig 4C), nearly no cells would have high activation in both environments. In this way for Fig 4B, the cell shown in one plot would not occur in the other plot. Thus we individually reordered highly activated aLB cells in each single plot. Instead, S5 Fig provides the unordered results of all 360 aLB cells in all testing environments.

*In Fig. 4C (left and middle), I understand that we should see a 10x10 matrix visualization, but we see an intermediate green color that seems misleading.*

The intermediate green band occurred as a colour gradient. As using colour gradient here is indeed not ideal to visualise the data, we revised this figure to show 10-by-10 discrete data points.

*In the discussion, the authors make the connection of their study with the insect central complex. Many observations have been made, in recent years, on the activity and learning of the drosophila central complex. This is especially true in the context of salient visual stimuli which are relevant for the discussion. The authors might want to explore this literature and see whether it is relevant for their study.*

The referee is right to point to this highly intriguing literature. We have endeavoured to expand the relevant discussion in the subsection *Model Predictions* of *Discussion* section. New citations have also been added in the same paragraph.

We would like to thank Reviewer 2 for his/her time and effort and the constructive review.

**Others**

*Have the authors made all data and (if applicable) computational code underlying the findings in their manuscript fully available?*

Animal behavioural data are within the Supporting Information files (S1 Dataset). Simulation results data are available on Kaggle after acceptance of the manuscript for publication:

[www.kaggle.com/chronowanderer/aLBcells-for-HDsystem-simulation-results](http://www.kaggle.com/chronowanderer/aLBcells-for-HDsystem-simulation-results)

Relevant computational modelling codes (in MATLAB 2017a) are available on GitHub after acceptance of the manuscript for publication:

[www.github.com/Chronowanderer/aLBcells-for-HDsystem](http://www.github.com/Chronowanderer/aLBcells-for-HDsystem)

and

[www.github.com/Bicanski/aLBcells-for-HDsystem](http://www.github.com/Bicanski/aLBcells-for-HDsystem)

Before publication acceptance, reviewers could obtain simulation results data from a temporary private link of Kaggle

[www.kaggle.com/dataset/7588ba1666595aed9895519b428744f68ee63bbfaff4b3837997cc62f8df1105](http://www.kaggle.com/dataset/7588ba1666595aed9895519b428744f68ee63bbfaff4b3837997cc62f8df1105)

Also, as no private link could be generated on Github, we here provide computational modelling codes (Codes.zip, not mentioned in the manuscript), of which the files submitted are merely for review and thus not for final publication.

We thank the editors for reconsidering our manuscript.

Yours sincerely,

Yijia Yan, Prof Neil Burgess, Dr Andrej Bicanski

**References**

Carpenter, G. A., & Grossberg, S. (1987). A massively parallel architecture for a self-organizing neural pattern recognition machine. *Computer Vision, Graphics and Image Processing*, *37*(1), 54–115. https://doi.org/10.1016/S0734-189X(87)80014-2

Coultrip, R., Granger, R., & Lynch, G. (1992). A cortical model of winner-take-all competition via lateral inhibition. *Neural Networks*, *5*(1), 47–54. https://doi.org/10.1016/S0893-6080(05)80006-1

Jacob, P. Y., Casali, G., Spieser, L., Page, H., Overington, D., & Jeffery, K. (2017). An independent, landmark-dominated head-direction signal in dysgranular retrosplenial cortex. *Nature Neuroscience*, *20*(2), 173–175. https://doi.org/10.1038/nn.4465

Kim, S., Jung, D., & Royer, S. (2020). Place cell maps slowly develop via competitive learning and conjunctive coding in the dentate gyrus. *Nature Communications*, *11*(1), 1–15. https://doi.org/10.1038/s41467-020-18351-6

Riesenhuber, M., & Poggio, T. (1999). Hierarchical models of object recognition in cortex. *Nature Neuroscience*, *2*(11), 1019–1025. https://doi.org/10.1038/14819

Rumelhart, D. E., & Zipser, D. (1985). Feature discovery by competitive learning. *Cognitive Science*, *9*(1), 75–112. https://doi.org/10.1016/S0364-0213(85)80010-0

Sayer, R. J., Friedlander, M. J., & Redman, S. J. (1990). The time course and amplitude of EPSPs evoked at synapses between pairs of CA3/CA1 neurons in the hippocampal slice. *Journal of Neuroscience*, *10*(3), 826–836. https://doi.org/10.1523/jneurosci.10-03-00826.1990

Sharp, P. E. (1991). Computer simulation of hippocampal place cells. *Psychobiology*, *19*(2), 103–115. https://doi.org/10.3758/BF03327179

Zénon, A., Hamed, S. Ben, Duhamel, J. R., & Olivier, E. (2009). Attentional guidance relies on a winner-take-all mechanism. *Vision Research*, *49*(12), 1522–1531. https://doi.org/10.1016/j.visres.2009.03.010
